# Transplantation of patient-derived adipose stem cells in YAC128 Huntington's disease transgenic mice

**DOI:** 10.1371/currents.RRN1183

**Published:** 2010-09-26

**Authors:** Wooseok Im, Soon-Tae Lee, Jeong Eun Park, hyun Jung Oh, Jiyoung Shim, Jiyeon Lim, Kon Chu, Manho Kim

**Affiliations:** ^*^Neurology, Seoul National University Hospital; ^†^Department of Neurology, Seoul National University Hospital, Seoul, South Korea and ^**^Department of Neurology, Seoul National University Hospital

## Abstract

Huntington’s disease (HD) is a genetic neurodegenerative disorder caused by abnormal expansion of CAG in the huntingtin gene. In R6/2 HD transgenic mice, human adipose-derived stem cells (ASCs) can slow disease progression via secretion of multiple paracrine growth factors. In order to prompt autologous ASCs transplantation in HD, we isolated ASCs from subcutaneous adipose tissues from a HD patient and a normal volunteer. ASCs were grown in two different types of stem cell culture media, EGM-2MV (endothelial growth medium-2 MV) or mesenchymal culture medium (MesenPRO). Cell-surface markers CD13, CD29, CD31, CD34, and CD44 were characterized by flow cytometry. BDNF, HGF, IGF, LIF, NGF, and VEGF expressions were determined by RT-PCR. Cell surface markers for HD ASCs were similar to those for normal ASCs. HD ASCs expressed multiple growth factors, and were similar to normal ASCs, except for NGF; however, they can be altered by culture medium. ASCs were transplanted in bilateral striata of 8 month-old YAC128 mice. At 12 months of age, normal ASCs reduced striatal atrophy, while HD ASCs failed to prevent it. Compared to the control YAC128 group, no improvement in Rotarod performance was observed in any of the transplanted YAC128 mice. However, when normal ASCs were transplanted at 12 months, Rotarod performance was maintained for 4 weeks, with detectable transplanted cells in the striatum and periventricular area. In summary, cultured HD patient-derived ASCs express multiple growth factors with the same cell surface markers as those of normal ASCs in vitro. The efficacy of ASCs transplantation in YAC128 transgenic models can be modified, depending on the time window.

## Introduction

Huntington’s disease (HD) is a progressive and heritable neurodegenerative disorder, which is characterized genetically by abnormal expansion of CAG repeats in the huntingtin gene [1] [2]. It leads to the progressive loss of medium-sized spiny neurons in striatum,which represent the largest population of degenerated neurons in HD [3] [4]. Recent progress in stem cell biology has demonstrated the possibility of replacement of lost neural cells by transplantation of stem or progenitor cells in animal models of HD [5] [6] [7]. However, limitations in the efficacy of stem cell transplantation must be overcome before clinical application of cell therapy in treatment of HD. Adipose-derived stem cells (ASCs) can be easily isolated from waste tissue remaining after liposuction, and have multi-potency for differentiation into several lineages, including adipocytes, bone, cartilage, skeletal muscle, endothelium, hematopoietic cells, and neuronal cells [8] [9] [10]. In addition, ASCs have the ability to secrete multiple anti-apoptotic growth factors, including granulocyte monocyte colony stimulating factor (GM-CSF), vascular endothelial growth factor (VEGF), hepatocyte growth factor (HGF), basic fibroblast growth factor (bFGF), and insulin-like growth factor-1 (IGF-1) [11] [12]. Therefore, ASCs offer a clinically feasible source of stem cells for autologous cell-based therapy. We recently reported that growth factors secreted from stem cells could alleviate neuronal damage in R6/2 mice [13]. Although we investigated the paracrine effects of ASC transplantation in short-lived R6/2 mice, there are some questions as to whether this effect can be sustained in a transgenic HD model with a longer life span, such as YAC128, which is characterized by neurodegeneration in the cortical and striatal regions [14]. In addition, the efficacy of autologous ASCs from HD patients is unknown.

In this study, we obtained ASCs from a HD patient and a normal volunteer, and characterized these cells by flow cytometry and RT-PCR. These ASCs were transplanted into bilateral striata of 8-month-old YAC128. Four months later, we investigated the efficacy of HD ASC transplantation by behavioral and histological analysis.

## Materials and methods

### ***Isolation and culture of human adipose-derived stem cells***

All procedures were approved by the institutional review board of Seoul National University Hospital. Subcutaneous adipose samples were acquired with donors’ consent. Human ASCs were cultured as previously described [11]. Briefly, subcutaneous adipose tissue was digested in collagenase type 1 solution (Invitrogen, Carlsbad, CA, USA) with vigorous agitation for 1 h at 37°C. Mature adipocyte fractions were separated from stromal fractions by centrifugation at room temperature at 400×g for 10 min. After centrifugation, stromal fractions (pellet) were treated with red blood cell lysis buffer for 10 min at room temperature, filtered through a 100-μm nylon mesh, and centrifuged at room temperature at 400×g for 10 min. The pellet was re-suspended and cultured in endothelial growth medium-2MV (EGM-2MV; Clonetics, Walkersville, MD, USA) or mesenchymal culture medium (MesenPRO, Invitrogen). EGM-2MV contains VEGF, bFGF, EGF, IGF-1, hydrocortisone, and ascorbic acid in EBM-2 with a 5% fetal bovine serum mixture and MesenPRO is a reduced serum (2%) medium that is specifically formulated to support the growth of human mesenchymal stem cells (MSCs) in culture.

Cells were passaged and used for *in vivo* transplantation at 3 passages. Prior to transplantation, cells were labeled with Vybrant™ DiI Cell-Labeling Solution (Invitrogen) in order to keep track of transplanted cells.

### ***Cell counting***

We measured growth curves of ASCs by counting the number of cells. ASCs from passage 3 (P3) were seeded into a 6-well plate at 5×10^4^ cells per well and counted by hemocytometer after detachment of cells with 0.25% trypsin-EDTA (Gibco-BRL).

### ***Flow cytometry analysis***

For analysis of phenotypes of ASCs, flow cytometry analysis was performed on these cells, which were grown in either EGM-2MV or MesenPRO. In brief, ASCs from the third passage culture were collected, washed with PBS, and stained with antibodies for 30 min in the dark. Antibodies were added in concentrations recommended by the manufacturer (20 μl/10^6^ cells). ASCs are known to be similar to bone marrow-derived mesenchymal stem cells (MSCs) [9] [15] [16] with CD13, CD29, and CD44 cell-surface markers, but not the hematopoietic marker, CD34, nor the endothelial marker, CD31 [17] [18] [19] [20]; therefore, we examined these 5 cell-surface markers. Mouse anti-human antibodies included PE-, PE-cy5 or FITC-conjugated and were purchased from BD Pharmingen: CD13 (555394), CD29 (559882), CD31 (55546), CD34 (560942), and CD44 (555478). All experiments were performed in triplicate.

### ***RNA preparation and reverse transcription-polymerase chain reaction (RT-PCR)***

ASCs secrete BDNF, HGF, IGF, LIF, NGF, and VEGF, which are major factors of the paracrine effect in cell therapy [11] [13] [21]. We compared expression of growth factors between HD and normal ASCs in EGM-2MV or MesenPRO culture media. Total RNA was isolated from ASCs at passage 3 using the QIAshredder and RNeasy kits (Qiagen, Valencia, CA, USA). RNA samples were reverse-transcribed using the iScript cDNA synthesis kit (Bio-Rad). RT-PCR analysis was validated for each mRNA and primers for RT-PCR were as follows: BDNF (-TGA GCC TGT GTG ACA GTA TT-, -CAG CCT TCT TTT GTG TAA CC-), HGF (-AGA TTG TTA TCG TGG GAA GAA TG-, -GAG CAT CAT CAT CTG GAT TT ), LIF (-AAC AAC CTC ATG AAC CAG AT-, -GTG CCA AGG TAC ACG ACT AT-), NGF (-GGA CGC AGC TTT CTA TCC TGG-, -CCC TCT GGG ACA TTG CTA TCT G-), VEGF (-GTG GAC ATC TTC CAG GAG TA-, -TCT GAC TTC ACA TTT GTT GT-), IGF-1 (-ATG TCC TCC TCG CAT CTC TTC-, -CCT GTA GTT CTT GTT TCC TGC-).

### ***YAC128 transgenic HD animal model and ASC transplantation***

We used transgenic HD mice of the YAC128 line and their WT littermates (Jackson Laboratories, Bar Harbor, ME, USA). The YAC128 mouse model of HD contains the entire human HD gene containing 128 CAG repeats [14]. The genotype was assessed using a PCR assay [22]. For ASC transplantation at 8 months of age, mice were anesthetized with an intraperitoneal injection of zoletile (40 mg/kg) and xylazine hydrochloride (4 mg/kg). In the ASCs-transplanted groups (YAC128-ASCs(HD)-EGM-2MV, YAC128-ASCs(HD)-MesenPRO, and YAC128-ASCs(normal)-EGM-2MV), 2 μl containing 5×10^5^ cells suspended in PBS were injected into the bilateral striatum using a 30-guage Hamilton syringe in a stereotaxic apparatus at the following coordinates: AP +0.38 mm, ML ±2.0 mm, and DV 3.5 mm from the Bregma. ASCs were slowly injected over a period of 5 min. The needle of the Hamilton syringe was left in position for another 5 min and then gently removed.  For ASC transplantation at 12 months of age, YAC128 mice were transplanted with ASCs(normal)-EGM-2MV, and untreated YAC128 and wild type were used as control groups. At 4 weeks, all mice were sacrificed for sampling of brains. All animal experiments and procedures were performed after receiving institutional approval and complied with the NIH Guide for the Care and Use of Laboratory Animals.

### ***Behavioral tests and weight measurement of YAC128 mice***

Rotarod performance of YAC128 mice was tested as previously described [23], with the modification that the speed was increased linearly from 4 to 40 rpm over a period of 3 min (San Diego Instruments, San Diego, CA, USA). In ASC transplantation at 8 months of age, 5-month old mice were pre-trained, with three trials per day for three consecutive days (total 9 trials). They were then tested once daily, also for three consecutive days (average of these 3 trials for each time) every 4 weeks until 12 months of age. Mice were weighed every four weeks from the 20th week to the 52nd week. For ASC transplantation at 12 months of age, mice were tested every week for 5 weeks. 

### ***Striatal analysis***

For measurement of the striatal volumes of mouse brain, sliced tissues were stained by Nissl solution at intervals of 400 μm thickness. Lesion areas in individual sections were traced and measured using Image-Pro Plus (Media Cybernetics, Silver Spring, MD, USA), and the lesion areas were integrated for calculation of striatal volumes, as described previously [24].

### ***Statistical analysis***

All values are expressed as mean ± SD. For intergroup comparisons, the Mann-Whitney *U *test was used, and in these cases, we did not specify the test. For nonparametric comparison among 3 unpaired groups, we first used Kruskall-Wallis analysis of variances. When *p *values from Kruskall-Wallis were below 0.05, the Mann-Whitney *U *test was further used for post hoc intergroup comparisons. A 2-tailed probability value below 0.05 was considered to indicate significance.

## **Results**

### ***Culture and characterizations of ASCs Surface phenotypes and growth factors expression***

Isolation and culture of ASCs from both a HD patient and a normal volunteer was successful. Morphologies of these two ASCs grown in EGM-2MV did not differ (Fig 1). The proliferation pattern of ASCs from a HD patient (HD ASCs) was similar to that of ASCs from a normal volunteer (normal ASCs). However, after 48 hours, HD ASCs showed a trend toward being slightly higher in number than normal ASCs (Fig 1). The doubling time was 21 hours. Both HD and normal ASCs expressed CD13+, CD29+, and CD44+, but neither CD31 nor CD34 (Fig 2). HD ASCs grown in a different culture media (MesenPRO) expressed equal cell surface marker patterns; CD13+, CD29+, CD31-, CD34-, and CD44+ (Fig 3).

RT-PCR analysis indicated that BDNF and VEGF mRNAs were increased in HD ASCs, compared to normal ASCs, whereas NGF mRNA was expressed only in normal ASCs. HGF and LIF mRNAs expression were similar in HD and normal ASCs. IGF mRNA was not expressed in either. MesenPRO media increased IGF and NGF mRNA in HD ASCs. ASCs showed different morphologies, with a more spindle-like appearance in MesenPRO media. The relative expression difference between culture conditions (MesenPRO vs. EGM-2MV) are summarised in figure 4. We tested both HD ASCs-(EGM-2MV) and HD ASCs-(MesenPRO) for transplantation in YAC128 mice.

**Figure:**
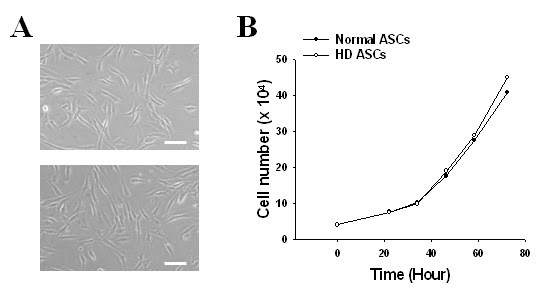
**Figure 1.** Morphology and growth curve of ASCs. (A) Normal ASCs (upper) and HD ASCs (lower) at 100× magnification. (B) Normal and HD ASCs grown in EGM-2MV were counted at 24 h, 36 h, 48 h, 60 h, and 72 h. HD ASCs showed a trend toward a slightly larger number of cells from 48 h. Bar=100 μm

**Figure:**
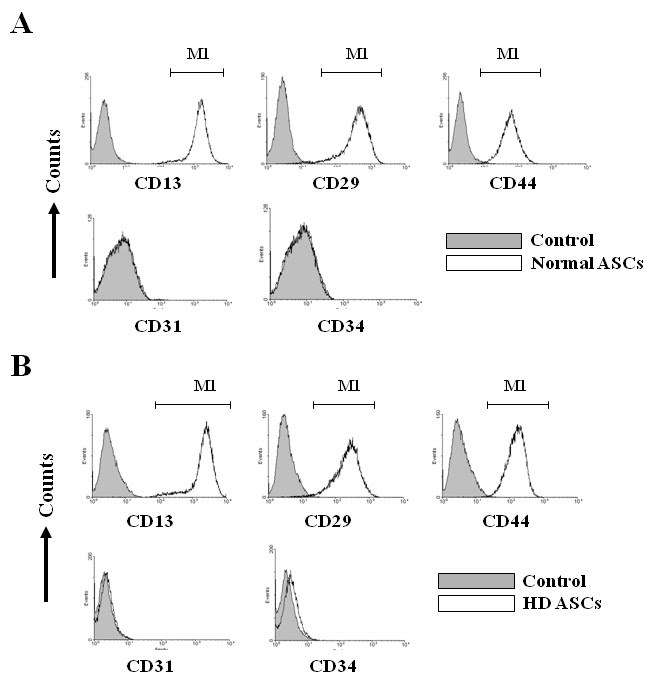
**Figure 2**. Flow cytometry analysis of cell surface markers on ASCs. In 3 passages, Normal ASCs (A) and HD ASCs (B) grown in EGM-2MV media were stained with a fluorescent-conjugated CD13, CD29, CD31, CD34, and CD44, and analyzed. Positive staining was defined as a fluorescence intensity greater than 99% of control. They showed CD13 (+), CD29 (+), and CD44 (+) markers, but did not have CD31or CD34. (“M1” bracket in histogram panel).  

**Figure:**
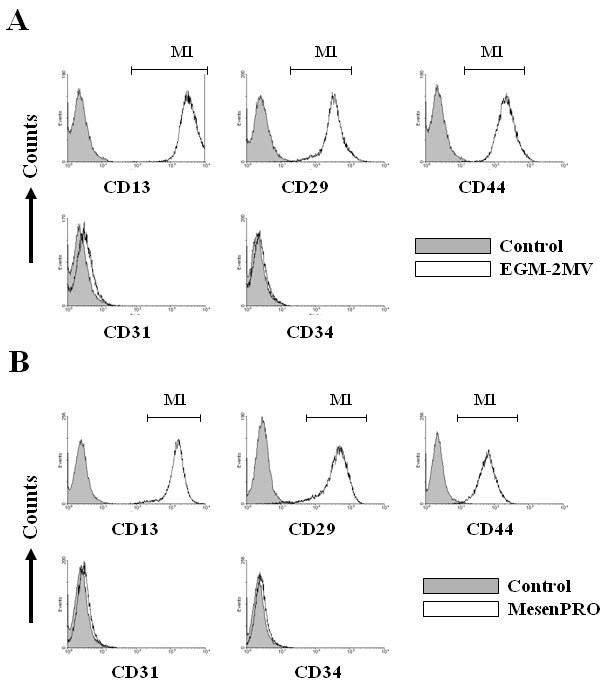
**Figure 3.** Comparison of cell surface markers on HD ASCs in two different culture media, EGM-2MV and MesenPRO. Markers in MesenPRO did not differ from EGM-2MV, expressing CD13+, CD29+, CD31-, CD34-, and CD44+. Fluorescence intensity greater than 99% of control was regarded as positive staining (“M1” bracket in histogram panel).

**Figure:**
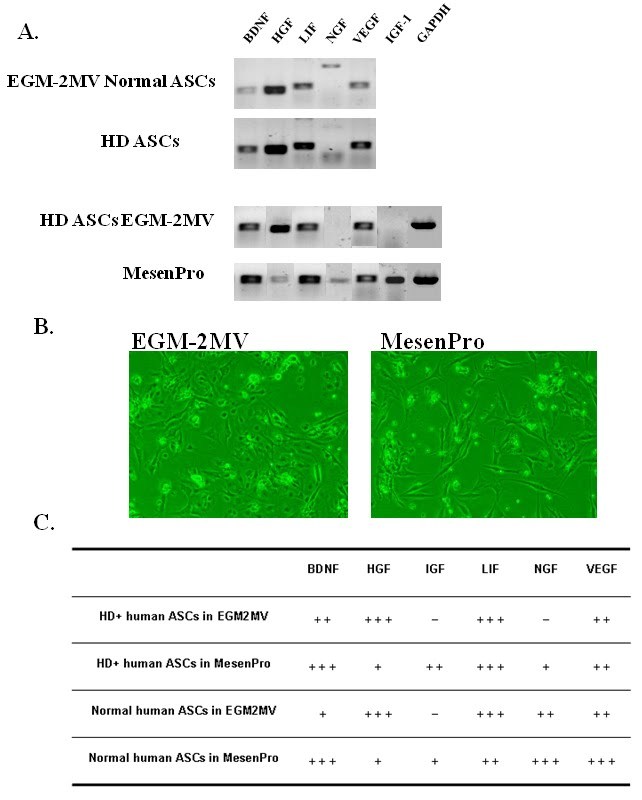
**Figure 4.** Expression of growth factor mRNAs in ASCs. (A). HD ASCs and normal ASCs in EGM-2MV culture conditions were compared. HD ASCs showed increased BDNF and LIF expression, whereas they did not show NGF expression. In HD ASCs, when the culture medium was changed from EMG-2MV to MesenPRO, expression profiles of growth factors were altered, expressing NGF and IGF-1, whereas HGF expression decreased. (B) Using two types of medium, ASCs morphology showed a more spindle-like appearance in MesenPRO. (C) The relative expression profile for each condition is summarized in the table. BDNF, brain-derived neurotrophic factor; HGF, hepatocyte growth factor; LIF, leukemia inhibitory factor; NGF, nerve growth factor; VEGF, vascular endothelial growth factor; IGF-1, insulin growth factor 1.

### ***Behavioral tests and histological analysis in ASCs-transplanted YAC128 mice***

We injected 8 month-old YAC128 mice with normal ASCs cultured in EGM-2MV [n=7; YAC128-ASC(normal)-EGM], HD ASCs cultured in EGM-2MV [n=7; YAC128-ASC(HD)-EGM], and HD ASCs cultured in MesenPRO [n=8; YAC128-ASC(HD)-MsesenPro]. In addition, untreated YAC128 (n=8; YAC128-control) and wild type (n=8, WT) were used as control groups. After 4 months, all mice were sacrificed for sampling of brains (Fig 5A). YAC128-control mice showed progressive decline in Rotarod performance. Compared to YAC128 control mice, YAC128 mice transplanted with either normal ASCs or HD ASCs showed no significant difference in Rotarod performance (p>0.05 in Kruskall-Wallis analysis of variances) (Fig 5B). In measurements of body weight, all experimental groups showed slow incremental tendency of body weights, while there was no significant difference in mean body weight among the groups (P>0.05 in Kruskall-Wallis analysis of variances) (Fig 5C). No differences in behavioral and body weight outcomes were observed as a result of ASCs culture conditions, i.e. EGM-2MV and MesenPRO media.  To investigate differences in striatal degeneration in YAC128 transplanted with HD ASCs, we analyzed the striatal volume of mice at 12 months of age by analysis of Nissl-stained sections. YAC128-control mice presented smaller striatal volume, compared to WT mice (Fig 6). YAC128-ASC(normal)-EGM mice showed larger striatal volume compared to YAC128-control mice (p<0.05), suggesting slowed striatal atrophy by ASC transplantation. However, YAC128-ASC(HD)-EGM and YAC128-ASC(HD)-MesenPRO mice showed smaller striatal volumes, compared to YAC128-control or YAC128-ASC(normal)-EGM mice (p<0.05). 

**Figure:**
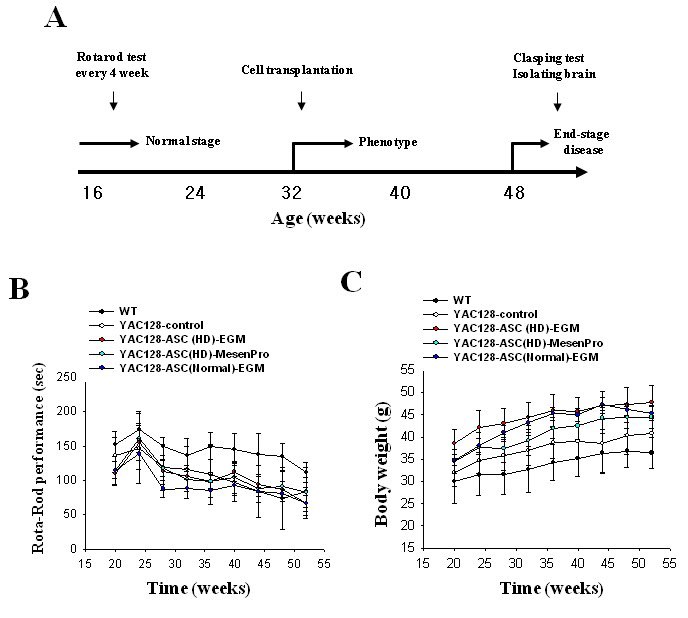
**Figure 5.** Experimental scheme and behavioral tests. The overall time frame and outline of experimental protocols is presented in (A). The YAC128-ASC group showed no difference in Rota-Rod performance, compared with the YAC128 control group (B). Mean body weights of YAC128-ASCs(normal)-EGM of the ASCs-injected groups were larger than those of YAC128-control during 28~48 weeks of age (C).

**Figure:**
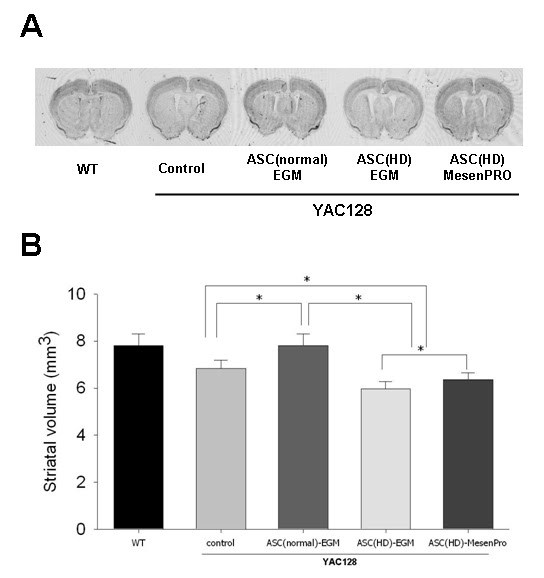
**Figure 6.** Striatal volume analysis. YAC128 mice brain showed brain atrophy, as indicated in representative Nissl-stained sections (A). Transplantation of normal ASC transplantation at the pre-phenotypic phase (8- month) reduced the striatal atrophy when measured at 12 months of age. However, transplantations of ASCs derived from HD patients were not effective in reducing striatal atrophy (B). *p < 0.05

### ***Transplantation during the phenotypic phase of YAC128 mice***

  To further determine the time window with regard to whether ASCs may have a paracrine effect during a specific period, we transplanted ASCs(normal)-EGM-2MV into the striatum of 12 month-old phenotypic stage YAC128 mice. This experiment included three groups: WT (n=3), YAC128-control (n=3), and YAC128 mice transplanted with ASC(normal) cultured in EGM-2MV (YAC128-ASC(normal)-EGM, n=4). While YAC128-control mice showed decline of Rotarod performance compared to WT mice during the 4-week observation period, YAC128-ASC(normal)-EGM mice presented better Rotarod performance at 1 and 4 weeks after transplantation, compared to YAC128-control mice (p<0.05 in Mann-Whitney *U *test after Kruskall-Wallis analysis among the three groups) (Fig 7A). No significant difference in measurements of striatal volume by Nissl staining were observed between the YAC128-ASC(normal)-EGM and YAC128-control groups (Fig 7B). DiI-labeled ASCs in YAC128-ASC(normal)-EGM were detected in the striatum and periventricular zone (Fig 7C).

**Figure:**
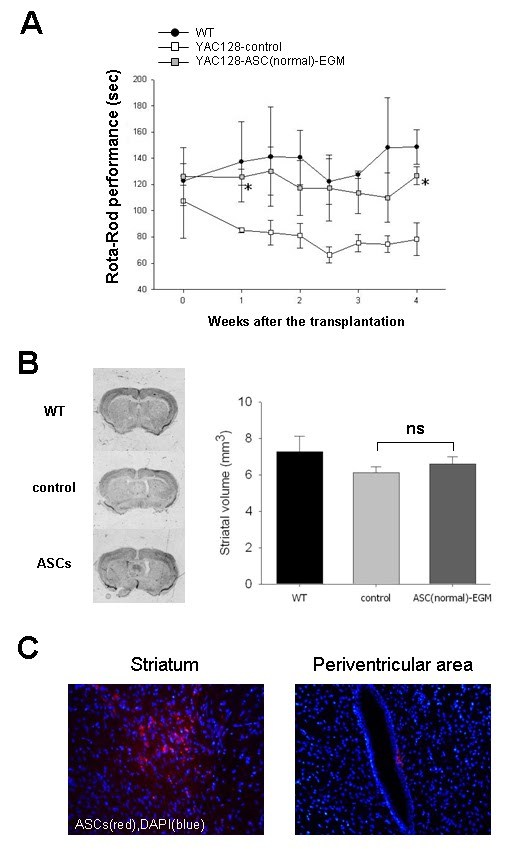
**Figure 7.** ASC transplantation at phenotypic phase (12-month). Unlike the 8-month protocol, ASC transplantation at 12 months of age increased Rota-rod performance at 1 and 4 weeks after transplantation, compared to YAC128-control mice (A). However, striatal volumes did not differ among the groups (B). In histologic sections, DiI-labeled ASCs (red) were detected in the striatum and periventricular area (C). *p < 0.05, ns=not significant.

## **Discussion**

In our experiment, we attempted to test the efficacy of patient-derived ASCs in YAC128 HD mice and to determine whether they can be used as a source of autologous stem cells for patients with HD. In this experimental design, HD ASCs showed little *in vivo* effect of transplantation; however, the cells also expressed paracrine growth factors, which are key mediators of the disease-modifying effects of ASCs transplantation in HD mice [13]. Cell surface markers of HD ASCs were similar to those of normal ASCs. The overall therapeutic outcome in YAC128 was not as effective as that of ASCs transplantation in R6/2 mice. While YAC128 mice showed slow disease progression for 12 months, R6/2 mice showed rapid phenotypes at 10 weeks of age [22]. Therefore, given that cell survival of ASCs is limited to a few weeks after transplantation [13], ASCs might have fewer effects on the long standing progression of the disease in YAC128 mice. 

In HD ASCs in EGM-2MV media, lowered NGF expression was noted in comparison to normal ASCs. Recent reports have suggested that stem cell therapy has focused on the paracrine effect rather than replacement of transplanted cells [24] [25]. Various growth factors secreted from ASCs, including VEGF, HGF, bFGF, and IGF-1, are associated with neuroprotective effects or enhancement of neurogenesis [11] [21] [26] [27] and FGF-2 and IGF-1 have proven to be beneficial in experimental models of HD [28] [29]. MesenPRO and EGM-2MV culture media showed different levels of growth factor expression in HD ASCs. MesenPRO media increased NGF and IGF-1 expression in HD-ASCs. However, transplantation of HD ASCs cultured from each medium did not show a difference under our experimental conditions. 

In a short-lived R6/2 mice study, behavioral assessment and histological analysis were performed within two months following transplantation, and approximately 13% of transplanted ASCs were detected at one month in our previous study [13].In this study, we were not able to detect HuN (human nuclei) antibody-positive cells in the ASC-transplanted brain. When mice were transplanted with DiI dye-labeled ASCs at 8 months, none of the cells were detected at 12 months as well, suggesting an extremely low % of cell survival. Thus, we further conducted ASCs transplantation in 12-month-old (phenotypic) YAC128 mice, and, 4 weeks later, we were able to detect DiI-labeled ASCs in the striatum and periventricular area. Moreover, significant differences in Rotarod performance were observed between the two transgenic groups.     Compared to WT mice, YAC128-control mice did not show definite attenuations of body weight. YAC128 mice transplanted with ASCs showed heterogenous body weight curves. Moreover, there was a difference in initial mean body weights at 20 weeks of age in our experiment. Therefore, analysis of the effect of transplantation on body weight in our study is limited.

In summary, our results show that HD ASCs have cell surface markers that are similar to those of normal ASCs. HD ASCs also have similar patterns of expression of growth factors, which can be modified, depending on the culture conditions. Although transplantation protocol at 8 months and behavioral assessment by 12 months appeared to be ineffective, there are time windows for testing the efficacy of ASCs in a YAC128 model. For an effective source of autologous stem cells from patients, development of a strategy to augment the paracrine effect in order to sustain a long period in vivo and to define a more appropriate time window is warranted.

## **Funding information**

 This work was supported by grants from CHDI, WCU-Neurocytomics and the Korea Health 21 R&D  Project (A092058).  

## **Competing interests**

The authors have declared that no competing interests exist.
